# Rebuttal to: Letter to the Editor. Re: “[An extensive dataset of handwritten central Kurdish isolated characters by R.M. Ahmed, T.A. Rashid, P. Fatah, A. Alsadoon & S. Mirjalili, Data in Brief, 2021, 39, 107479]”

**DOI:** 10.1016/j.dib.2024.110072

**Published:** 2024-01-22

**Authors:** Rebin M. Ahmed, Tarik A. Rashid

**Affiliations:** a IT Department, Tishk International University, Erbil, Iraq; b Computer Science and Engineering Department, University of Kurdistan-Hawlêr, Erbil, Iraq

## 1 Overview

In this response, we address feedback received in a Letter to the Editor regarding the dataset presented in the original article, “An Extensive Dataset of Handwritten Central Kurdish Isolated Characters” [1]. The original article focuses on the subject area of Kurdish handwriting recognition, presenting an extensive dataset of isolated characters. The dataset's key points involve the compilation process, potential biases, representation, and description within the original article, as well as considerations of data reusability. The Letter to the Editor highlights specific concerns related to the dataset, including discrepancies in the representation of certain letters (“ژ,” “س,” and “ش”), labeling errors, and inaccuracies in reported proportions and percentages in the accompanying tables. This response addresses these concerns, providing clarifications and actions taken to rectify the issues, with a commitment to maintaining high standards of accuracy and data quality.

## 2 Details

We would like to express our sincere appreciation for the author's detailed letter regarding the dataset for Kurdish handwriting recognition, An Extensive Dataset of Handwritten Central Kurdish Isolated Characters [1].

We acknowledge and value the author's efforts in highlighting discrepancies and errors in the published paper and dataset, and we understand the importance of maintaining data accuracy and integrity in research.

After a thorough review of the issues authors raised, we have taken the necessary actions to address them. Here are the specific clarifications:

1. Letter “ژ” (letter 14)

The authors mention 11 additional images for the letter “ژ” beyond the reported count. Upon further investigation and review of data collection notes, it was discovered that participants had written incorrect letters on the last set of forms for this letter. These incorrect images were initially excluded from the review process, but they were inadvertently retained in the folder. Therefore, the folder contains empty images that do not represent any letters and have been removed from the updated version of the dataset.

2. Letter “س” (letter 15)

Due to a minor error in the labeling process, an extra image was added to set 01 for the letter “س,” increasing the total image count from 126 to 127. This error has been corrected in the updated version of the dataset.

3. Letter “ش” (letter 16)

The final batch of data for the letter “ش” was inadvertently omitted from the published version of the dataset. Although the form was scanned, preprocessed, and labeled, it was not included in the original release. Consequently, the published table incorrectly reports that set 10 comprises 126 images, when, in fact, that set was not included in the previously published dataset. This error has been corrected in the updated version of the dataset.

Regarding errors in tables, we acknowledge miscounts in the proportions and percentages due to human error during data collection and labeling. We have carefully reviewed and corrected these errors in the updated dataset and the corresponding tables. The corrected tables (Table 1: Number and Percentage of Collected Letters and Table 2: Sets of Data Collection) accurately reflect the actual proportions and percentages of each class. You can access the updated dataset and corrected tables.

While we appreciate the meticulous examination and dedication invested in identifying discrepancies and errors within the reported numerical data, we respectfully contend that these anomalies do not warrant classification as major issues. As indicated in Table 1, the alterations in percentage are minimal, with a negligible 0.01 variation observed in both instances. Notably, the percentage for the letter ‘ش’ remains unchanged.

## 3 Update

After a thorough examination of your correspondence, we have diligently revised our dataset on Mendeley Data, which is accessible through the provided link [https://data.mendeley.com/datasets/f8z9jts5nb/3] [2]. Subsequently, a new version has been disseminated, encompassing the modifications delineated in the details section of this rebuttal letter, along with the pertinent adjustments integrated into Tables 1 and 2.

**Table 1:** Number and percentage of letters (ژ and س) before and after the review.

Order	ID	Letter	Number of images (Before review)	Number of images (After review)	Percentage (Before review)	**Percentage (After review)**
14	14	ژ	1123	**1126**	2.74%	**2.75%**
15	15	س	1107	**1108**	2.70%	**2.71%**
Total number			40,940	**40,944**	100%	100%

**Table 2:** Sets of data collection with updated numbers.

ID	Letter	Set 1	Set 2	Set 3	Set 4	Set 5	Set 6	Set 7	Set 8	Set 9	Set 10	Total
14	ژ	126	0	126	126	126	0	0	126	126	**370**	**1126**
15	س	**127**	126	126	99	126	126	126	126	126	0	**1108**
	Total	**4411**	3528	4410	4158	4158	4031	3905	4536	4284	**3523**	**40,944**

Table 1 exhibits the updated percentages for letters (ژ and س). For letter ژ, the total number was adjusted from 1123 to 1126 after the removal of empty images and a recount of the total images. Similarly, the count for letter س was amended from 1107 to 1108 images.

Table 2 presents the updated numbers for both Set 1 and Set 10, along with the overall image count. Notably, the number of images from Set 1 for letter س was modified from 126 to 127, resulting in the total changing from 4410 to 4411. Additionally, Set 10 experienced adjustments, with the number of images changing from 367 to 370 and the total for Set 10 shifting from 3520 to 3523 images. Consequently, the overall total number of images was revised from 40,940 to 40,944.

## Ethics Statement

This letter adheres to the Data in Brief guidelines and does not constitute original research. As such, it does not involve human subjects, animal studies, or data gathered from social media platforms. Therefore, no ethical approval, informed consent, or ethical considerations relevant to these aspects are applicable to this communication.

## CRediT authorship contribution statement

**Rebin M. Ahmed:** Conceptualization, Methodology, Writing – review & editing. **Tarik A. Rashid:** Supervision, Validation, Writing – review & editing.

## References

[1] Ahmed R.M., Rashid T.A., Fatah P., Alsadoon A., Mirjalili S. (2021). An extensive dataset of handwritten central Kurdish isolated characters. Data Br..

[2] Ahmed, Rebin M., Rashid T., Fattah P. (2023). An extensive dataset of handwritten central Kurdish isolated characters. Mendeley Data.

